# *Prevotella jejuni* sp. nov., isolated from the small intestine of a child with coeliac disease

**DOI:** 10.1099/ijs.0.052647-0

**Published:** 2013-11

**Authors:** Maria E. Hedberg, Anne Israelsson, Edward R. B. Moore, Liselott Svensson-Stadler, Sun Nyunt Wai, Grzegorz Pietz, Olof Sandström, Olle Hernell, Marie-Louise Hammarström, Sten Hammarström

**Affiliations:** 1 Department of Clinical Microbiology, Immunology, Umeå University, SE-90187 Umeå, Sweden; 2 CCUG – Culture Collection University of Gothenburg, Department of Clinical Bacteriology, Sahlgrenska University Hospital, SE-41345 Göteborg, Sweden; 3 Department of Infectious Diseases, Sahlgrenska Academy of the University of Gothenburg, SE-40530 Göteborg, Sweden; 4 Department of Molecular Biology, Umeå University, SE-90187 Umeå, Sweden; 5 Department of Clinical Sciences, Pediatrics, Umeå University, SE-90187 Umeå, Sweden

## Abstract

Five obligately anaerobic, Gram-stain-negative, saccharolytic and proteolytic, non-spore-forming bacilli (strains CD3 : 27, CD3 : 28^T^, CD3 : 33, CD3 : 32 and CD3 : 34) are described. All five strains were isolated from the small intestine of a female child with coeliac disease. Cells of the five strains were short rods or coccoid cells with longer filamentous forms seen sporadically. The organisms produced acetic acid and succinic acid as major metabolic end products. Phylogenetic analysis based on comparative 16S rRNA gene sequence analysis revealed close relationships between CD3 : 27, CD3 : 28^T^ and CD3 : 33, between CD3 : 32 and *Prevotella histicola* CCUG 55407^T^, and between CD3 : 34 and *Prevotella melaninogenica* CCUG 4944B^T^. Strains CD3 : 27, CD3 : 28^T^ and CD3 : 33 were clearly different from all recognized species within the genus *Prevotella* and related most closely to but distinct from *P. melaninogenica*. Based on 16S rRNA, RNA polymerase β-subunit (*rpoB*) and 60 kDa chaperonin protein subunit (*cpn60*) gene sequencing, and phenotypic, chemical and biochemical properties, strains CD3 : 27, CD3 : 28^T^ and CD3 : 33 are considered to represent a novel species within the genus *Prevotella*, for which the name *Prevotella jejuni* sp. nov. is proposed. Strain CD3 : 28^T^ ( = CCUG 60371^T^ = DSM 26989^T^) is the type strain of the proposed novel species. All five strains were able to form homologous aggregates, in which tube-like structures were connecting individual bacteria cells. The five strains were able to bind to human intestinal carcinoma cell lines at 37 °C.

Coeliac disease (CD) is an immune-mediated enteropathy with a multifactorial aetiology. Early childhood infections have been shown to be a risk factor for CD (Myléus *et al.*, 2012). Also, the jejunal microbiota is considered to play a role in the pathogenesis of CD (Olivares *et al.*, 2013). This is supported by epidemiological data from Sweden showing that childhood CD has features of an infectious disease with a peak incidence between 1985 and 1996 in children younger than 2 years of age, a period referred to as ‘the Swedish CD epidemic’ (Ivarsson *et al.*, 2000). A similar increase in incidence was seen later, during 2001–2004 (Olsson *et al.*, 2008; Namatovu, F. *et* *al.*, unpublished data). After both peaks, incidence returned to normal. We have shown that CD patients born during ‘the Swedish CD epidemic’ had a significant enrichment of mucosa-associated rod-shaped bacteria of the order *Clostridiales*, and genera *Prevotella* and *Actinomyces* in the jejunum (Forsberg *et al.*, 2004; Ou *et al.*, 2009). Recently, we characterized a novel species of a new genus, *Lachnoanaerobaculum umeaense*, that had been isolated from the jejunal mucosa of a child born during ‘the Swedish CD epidemic’ (Hedberg *et al.*, 2012). We assumed that this bacterium corresponded to the prevalent bacteria of the order *Clostridiales* we had previously reported (Ou *et al.*, 2009). To further characterize the microbiota of the small intestine of children with CD born during the first epidemic we have now studied isolates of the genus *Prevotella*.

At the time of writing, 48 species of the genus *Prevotella* have been described (Euzéby, 2013). The vast majority were isolated from humans, with the oral cavity being the main source (Dewhirst *et al.*, 2010). However, *Prevotella* species have also been isolated from faeces (Hayashi *et al.*, 2007), the female genital tract, skin and respiratory tract, and from the rumen and hindgut of non-human mammals (Alauzet *et al.*, 2010). Until now, no species of the genus *Prevotella* from the human small intestine had been characterized. Species of the genus *Prevotella* are generally considered to be non-pathogenic or opportunistic pathogens. However, they have been shown to be involved in serious infections, and virulence factors such as haemolysins, haemagglutinins, fimbrial adhesins, proteases and phospholipases have been demonstrated in strains of several species (Alauzet *et al.*, 2010).

This study describes the phenotypic and genotypic characterization of strains CD3 : 27, CD3 : 28^T^ and CD3 : 33, representing isolates of a novel species, CD3 : 32, probably a strain of *Prevotella histicola* (Downes *et al.*, 2008), and CD3 : 34, probably a strain of *Prevotella melaninogenica* (Shah & Collins, 1990). Additionally, we describe the phylogenetic relationships between the five isolates and other members of the genus *Prevotella*, based upon comparative 16S rRNA gene sequence analyses. Moreover, the five isolates have been subjected to whole genome sequencing (WGS) using 454 pyro-sequencing technology (GS Junior; Roche Diagnostics), and the sequences of the genes encoding chaperonin 60 (*cpn60*) and DNA-directed RNA polymerase subunit-β (*rpoB*) have also been compared.

The five strains were isolated from a biopsy of the proximal small intestine of a girl with CD, born in 1995, i.e. during the 1985–1996 Swedish CD epidemic. She was on a gluten-free diet when the biopsy was taken at the Department of Paediatrics, Umeå University Hospital, Umeå, in 2007. Informed consent was obtained from her parents. The study was approved by the local Research Ethics Committee of the Faculty of Medicine (Um dnr: 96-304 and 04-156). The biopsy was weighed, homogenized and serially diluted ten-fold in Fastidious Anaerobe Broth medium (Lab M) and immediately plated onto selective and non-selective agar media. All *Prevotella* strains were primarily isolated on blood agar plates [Columbia Blood Agar Base (Acumedia), supplemented with 5 % defibrinated horse blood]. *P. histicola* CCUG 55407^T^, *P. melaninogenica* CCUG 4944B^T^ and *Prevotella stercorea* CCUG 55595^T^ were obtained from the Culture Collection University of Gothenburg (CCUG; http://www.ccug.se).

Pure cultures of the five strains grew well on blood agar plates and in Brucella broth (BBL) supplemented with vitamin K (1 µg ml^−1^) and haemin (5 µg ml^−1^) under an anaerobic atmosphere (10 % H_2_, 5 % CO_2_ in N_2_) at 37 °C.

Colony morphologies and the results of presumptive identification tests by diagnostic discs (Jousimies-Somer *et al.*, 2002) were examined on blood agar plates after incubation for 3–5 days. None of the five strains grew in the presence of oxygen and they should be considered strictly anaerobic. Growth was improved and pigmentation and haemolytic activity increased if the atmosphere contained 10 % CO_2_ and 5 % H_2_ as compared with standard conditions. The appearance of the colonies of the five strains differed: isolate CD3 : 27 had circular, raised, convex, weakly to moderately pigmented and strongly haemolytic colonies; CD3 : 28^T^ and CD3 : 33 had circular, convex, weakly pigmented, weakly haemolytic colonies; CD3 : 32 had circular, slightly raised and brown-reddish pigmented colonies with a shiny ‘wet’ appearance. Moreover, the centres of the colonies of CD3 : 32 were darker than the outer part. Colonies of CD3 : 34 were similar to those of CD3 : 28^T^ and CD3 : 33, but with a surface appearing ‘drier’ (Fig. S1 available in IJSEM Online).

Light microscopy after Gram staining, dark field microscopy, scanning electron microscopy (SEM) and transmission electron microscopy (TEM) were used to investigate cell morphologies. Cells of the five strains were rod-shaped, 0.7×0.8–2 µm in size, occurring most often as short rods or as coccoid cells, with longer filamentous forms (>10 µm) seen sporadically. All five strains were Gram-stain-negative and lacked spores. SEM revealed that all five strains, particularly if grown on agar medium, as opposed to in liquid culture, formed large aggregates of bacterial cells connected to each other by multiple, thin, strait, rod-shaped structures (Fig. 1a–c). Cells of strains CD3 : 27, CD3 : 28^T^ and CD3 : 33 showed a similar degree of interconnectivity. Outer membrane vesicles were frequently observed. Analysis of thin sections of the aggregates by TEM suggested that the rod-shaped structures were hollow, characterized as tubes connecting cells to each other (Fig. 1d).

**Fig. 1.**
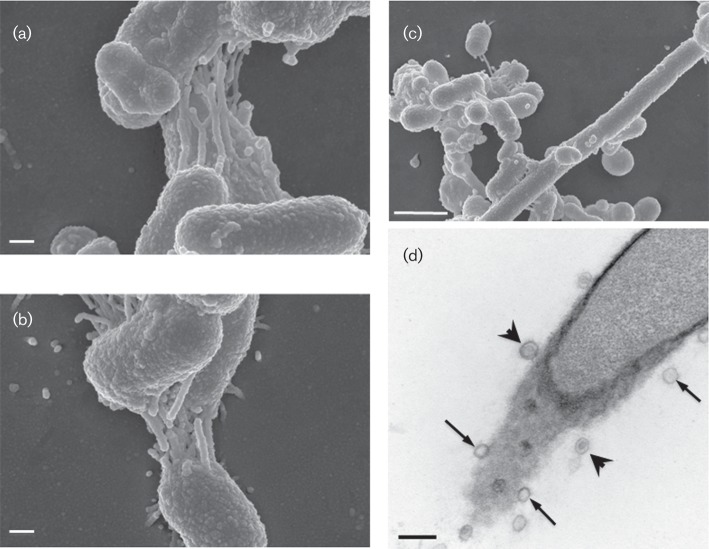
Scanning electron micrographs showing surface structures of cells of *P. jejuni*, strains CD3 : 27 (a) and CD3 : 28^T^ (b) and *P. histicola* strain CD3 : 32 (c). (d) Transmission electron micrograph of a cell of strain *P. jejuni* CD3 : 33; arrows indicate cross-section of the tube-like structures shown in (a)–(c) and arrowheads indicate outer membrane vesicles. Bars, 0.2 µm (a, b, d); 1 µm (c).

All five strains exhibited a temperature optimum for growth at 37 °C. The optimal pH for growth was 6–7 with reduced growth at pH 5.5 and 7.5. Motility was not observed. All five strains were haemolytic and produced NH_3_. Growth on glucose as the sole carbon source yielded acetic acid, succinic acid and small amounts of isovaleric acid for strains CD3 : 27, CD3 : 32 and CD3 : 34, and acetic acid and succinic acid for strains CD3 : 28^T^ and CD3 : 33.

The nucleotide sequences of the 16S rRNA genes of strains CD3 : 27, CD3 : 28^T^, CD3 : 33, CD3 : 32 and CD3 : 34 were determined by primer walking, covering the gene, and by cloning and sequencing of PCR amplification fragments also covering the gene (Hedberg *et al.*, 2012). These sequences were subsequently confirmed by genomic sequencing, allowing us to establish that there was only one copy of the 16S rRNA gene per genome. Other 16S rRNA gene sequences for comparative analyses were retrieved from the NCBI database (Sayers *et al.*, 2010). Strains CD3 : 27, CD3 : 28^T^ and CD3 : 33 shared >99.8 % 16S rRNA gene sequence similarity with each other and 98.1–98.3 % similarity with *P. melaninogenica* CCUG 4944B^T^ (AY323525), *P. histicola* CCUG 55407^T^ (AB547685), N 12-20 (EU126662), CD3 : 34 and CD3 : 32, and 97.3–97.7 % similarity with *Prevotella veroralis* CCUG 15422^T^ (AY836507). Strain CD3 : 32 was related most closely to *P. histicola* (AB547685 and EU126662) showing >99.6 % sequence similarity. Strain CD3 : 34 showed 99.8 % sequence similarity to *P. melaninogenica* (AY323525 and NC-014370). Fig. 2 shows the phylogenetic tree reconstructed using the maximum composite likelihood model based on 16S rRNA gene sequences. Strains CD3 : 27, CD3 : 28^T^ and CD3 : 33 formed a separate group distinct from recognized species of the genus *Prevotella* while strain CD3 : 32 clustered with *P. histicola* and strain CD3 : 34 with *P. melaninogenica*.

**Fig. 2.**
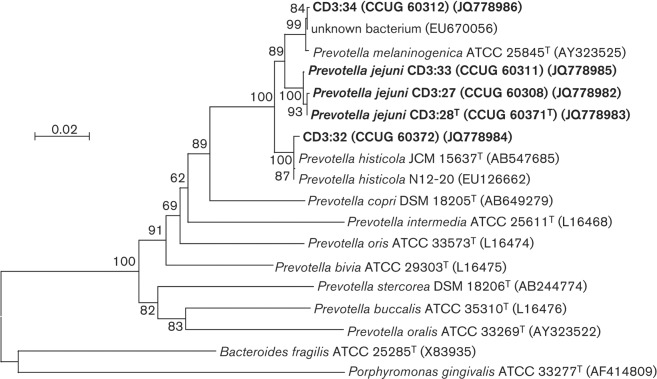
Phylogenetic tree based on 16S rRNA gene sequences showing the relationships between strains CD3 : 27, CD3 : 28^T^ and CD3 : 33 and related species. The 16S rRNA gene sequence of *Porphyromonas gingivalis* ATCC 33277^T^ served as an outgroup. Bar, 0.02 substitutions per nucleotide position.

Genomic DNA–DNA reassociation analysis was carried out using the hybridization protocols described by Urdiain *et al.* (2008). Strain CD3 : 28^T^ hybridized to a high level (95–112 %) with strains CD3 : 27 and CD3 : 33, confirming that these three strains belong to the same species. The level of hybridization between strain CD3 : 28^T^ and *P. histicola* CCUG 55407^T^, *P. melaninogenica* CCUG 4944B^T^, *Prevotella scopos* CCUG 57945^T^ and *P. veroralis* CCUG 15422^T^ was below 43 %. Levels of hybridization between strain CD3 : 28^T^ and strains CD3 : 32 and CD3 : 34 were 49 and 59 % respectively. The level of hybridization between *P. melaninogenica* CCUG 4944B^T^ and strain CD3 : 34 was high (104 %), while that between strain CD3 : 34 and strain CD3 : 28^T^ was 51 %. *P. melaninogenica* hybridized to a low level (30 %) with *P. histicola* CCUG 55407^T^. The coefficient of variation was less than 5.5 %. As the genomic DNA hybridization values were well below 70 % for strains CD3 : 27, CD3 : 28^T^ and CD3 : 33 on the one hand and strains CD3 : 32 or CD3 : 34 on the other, the strains can be considered to represent different species (Stackebrandt & Goebel, 1994).

To shed further light on whether CD3 : 27, CD3 : 28^T^ and CD3 : 33 should be considered as strains of the same novel species we compared the nucleotide sequences of the *rpoB* and *cpn60* genes (Alauzet *et al.*, 2010; Sakamoto & Ohkuma, 2010). Similarly, we compared strain CD3 : 32 with *P. histicola* and strain CD3 : 34 with *P. melaninogenica*. The *rpoB* and *cpn60* (3810 and 1626 nt respectively) gene sequences were 100.0 % identical between strains CD3 : 27, CD3 : 28^T^ and CD3 : 33. Sequence similarity between CD3 : 32 and *P. histicola* F0411 was 99.3 % for *rpoB* and 98.7 % for *cpn60*. Strain CD3 : 34 and *P. melaninogenica* CCUG 4944B^T^ shared 98.3 % *rpoB* gene sequence similarity and 97.7 % *cpn60* gene sequence similarity.

The sizes of the genomes and the DNA G+C contents of the five strains were determined from WGS data (Table 1). Strains CD3 : 28^T^ and CD3 : 33 had almost the same genome size, 3.81×10^6^ and 3.80×10^6^ bp, respectively, while CD3 : 27 had a size of 3.68×10^6^ bp. The genome of strain CD3 : 32 had a size of 3.20×10^6^ bp, larger than that of the closely related *P. histicola* F0411 (2.99×10^6^ bp). The genome size of strain CD3 : 34 was 3.27×10^6^ bp, about 102×10^3^ bp larger than that of *P. melaninogenica* CCUG 4944B^T^. The DNA G+C contents of the strains grouped together, in that strains CD3 : 27, CD3 : 28^T^ and CD3 : 33 had values of 41.7–41.8 mol%, CD3 : 32 and *P. histicola* F0411 had values of 41.1 and 41.2 mol%, respectively, and CD3 : 34 and *P. melaninogenica* CCUG 4944B^T^ values of 40.7 and 41.0 mol%, respectively.

**Table 1.** Genome size and DNA G+C content of *Prevotella jejuni* sp. nov., and the other two *Prevotella* isolates from human small intestine compared with *P. histicola* and *P. melaninogenica*

Isolate	Genome size (×10^6^ bp)	G+C content (mol%)
*P. jejuni* sp. nov. CD3 : 27 (CCUG 60308)	3.676	41.84
*P. jejuni* sp. nov. CD3 : 28^T^ (CCUG 60371^T^)	3.808	41.73
*P. jejuni* sp. nov. CD3 : 33 (CCUG 60311)	3.801	41.73
CD3 : 32 (CCUG 60372)	3.200	41.10
*P. histicola* F0411*	2.987	41.18
CD3 : 34 (CCUG 60312)	3.271	40.68
*P. melaninogenica* CCUG 4944B^T^*	3.169†	40.98

* From the NCBI sequence database.

† Chromosome I plus chromosome II.

Cellular fatty acid (CFA) methyl ester analyses were performed using a standardized protocol (http://www.ccug.se/pages/CFA_method_2008 and as detailed by Hedberg *et al.*, 2012). Strains were grown anaerobically (10 % H_2_, 5 % CO_2_ in N_2_), using chocolate agar as culture medium at 37 °C, and harvested after 48 h. CFAs were extracted and saponified by mild alkaline methanolysis and released fatty acids were methylated. CFAs were identified and quantified by GC (Hewlett Packard HP 5890). Retention times of CFA peaks were converted to equivalent chain-length values and the relative amount (w/w) of each fatty acid was expressed as a percentage of the total fatty acids in the profile of the respective strain (Table S1). The major CFAs detected in strains CD3 : 27, CD3 : 28^T^, CD3 : 33, CD3 : 32 and CD3 : 34 were iso-C_15 : 0_, anteiso-C_15 : 0_, C_16 : 0_, C_18 : 2_ω6,9*c*/anteiso-C_18 : 0_ and iso-C_17 : 0_ 3-OH. These five CFAs occurred in approximately the same relative amounts in the five strains with anteiso-C_15 : 0_ accounting for 38.5–42.5 % of the total CFAs. Interestingly, strains CD3 : 27, CD3 : 28^T^, CD3 : 33, CD3 : 32 and CD3 : 34 were more similar to each other than were CD3 : 32 to *P. histicola* CCUG 55407^T^ or CD3 : 34 to *P. melaninogenica* CCUG 4944B^T^ (Table S1). The similarities between the five jejunal isolates, although representing three different species, are perhaps a reflection of the fact that they were isolated from the same organ of one individual.

Analysis of metabolic and biochemical characteristics (rapid ID 32A, API 20A and APIZYM; bioMérieux) showed that the five strains are saccharolytic and proteolytic (Table S2). Strains CD3 : 27, CD3 : 28^T^ and CD3 : 33 demonstrated an almost identical pattern of biochemical characteristics. The only difference observed was that strain CD3 : 33 had α-galactosidase activity, while the other two strains did not. CD3 : 32 and *P. histicola* CCUG 55407^T^ showed an identical pattern of biochemical characteristics and the same was true for the comparison between CD3 : 34 and *P. melaninogenica* CCUG 4944B^T^. Sialidase activity was detected using 2′-(4-methylumbelliferyl)α-d-*N*-acetylneuraminic acid as substrate (Moncla & Braham, 1989). All strains produced sialidase except CD3 : 32 and *P. histicola* CCUG 55407^T^.

By disc diffusion it was shown that all five isolates and *P. histicola* CCUG 55407^T^ were resistant to vancomycin (5 µg) but susceptible to kanamycin (1 mg), colistin (10 µg) (Oxoid) and bile (1000 µg) (Oxgall tablets; Rosco Diagnostica), whereas *P. melaninogenica* CCUG 4944B^T^ was resistant to vancomycin and kanamycin but susceptible to colistin and bile. *P. stercorea* CCUG 55595^T^ was resistant to kanamycin and colistin but susceptible to bile and unexpectedly also susceptible to vancomycin (Jousimies-Somer *et al.*, 2002). Because the bacteria were isolated from the small intestine adjacent to the bile duct, susceptibility to bile was investigated further using an agar dilution technique. A stock solution containing 320 mM synthetic bile acids (taurocholate, 134.4 mM; taurochenodeoxycholate, 83.2 mM; glycocholate, 70.4 mM; glycochenodeoxycholate, 32 mM) yielded final concentrations of 0.125–16 mM bile acids in the assay. Interestingly, growth and haemolytic activity of all five jejunum isolates were stimulated at low concentrations of bile (0.5–1.5 mM) compared with medium without bile, while growth was inhibited at higher bile concentrations (2–8 mM).

Susceptibility to penicillin G was tested using MIC Evaluator Strips (Oxoid). Strains CD3 : 32, CD3 : 34 and *P. histicola* CCUG 55407^T^ were resistant (MIC >32 µg ml^−1^). The other strains were susceptible to penicillin G, with MICs ranging from 0.003 to 0.015 µg ml^−1^. According to the nitrocefin disc test (Remel), strains CD3 : 32, CD3 : 34 and *P. histicola* CCUG 55407^T^ produce β-lactamase. WGS revealed the presence of the *cfxA* β-lactamase gene in strains CD3 : 32 and CD3 : 34, but not in *P. histicola* F0411, the only other *P. histicola* isolate that has been sequenced so far, or *P. melaninogenica* CCUG 4944B^T^. Strains CD3 : 32 and CD3 : 34 shared 100 and 99 % *cfxA* gene sequence similarity with *Prevotella marshii* CCUG 50419^T^, respectively.

The abilities of strains CD3 : 27, CD3 : 28^T^, CD3 : 33, CD3 : 32, CD3 : 34, *P. histicola* CCUG 55407^T^ and *P. melaninogenica* CCUG 4944B^T^ to agglutinate human erythrocytes was investigated. Strains CD3 : 27, CD3 : 28^T^ and CD3 : 33 strongly agglutinated human O and AB erythrocytes; there was no difference in the strength of the agglutination reaction between the three strains, nor was there a difference in their ability to agglutinate AB versus O red blood cells. Strain CD3 : 34 showed a weak agglutination reaction while strains CD3 : 32, *P. histicola* CCUG 55407^T^ and *P. melaninogenica* CCUG 4944B^T^ were negative. The finding that some strains of *P. melaninogenica* are able to weakly agglutinate red blood cells (Haraldsson *et al.*, 2005) is in agreement with our results.

To confirm that the five jejunal isolates were able to bind to intestinal epithelial cells, binding of PKH-2 fluorescence dye-labelled bacteria to PKH-26 fluorescence dye-labelled intestinal epithelial cells was studied by flow cytometry (Hara-Kaonga & Pistole, 2007). Binding was evaluated after incubation at 37 °C and at 4 °C for 1 h. The cell lines were T84 (colon carcinoma), LS174T (colon carcinoma), HT29 (small intestine-like carcinoma) and Int407 (fetal small intestine epithelial cells), all obtained from the American Type Culture Collection (Rockville, MD). At 37 °C, all five isolates were able to bind to the four cell lines with two exceptions: strains CD3 : 27 and CD3 : 28^T^ did not bind to LS174T cells (Table S3).

We conclude that strains CD3 : 27, CD3 : 28^T^ and CD3 : 33 represent a novel species of the genus *Prevotella*, for which the name *Prevotella jejuni* sp. nov. is proposed, that CD3 : 32 is a strain of *P. histicola* and that CD3 : 34 is a strain of *P. melaninogenica*. The latter two jejunal isolates have larger genome sizes than the corresponding previously characterized strains. All five jejunal isolates are able to bind to human intestinal epithelial cells.

## Description of *Prevotella jejuni* sp. nov.

*Prevotella jejuni* (je.ju′ni. L. gen. n. *jejuni* of or from the jejunum, referring to the isolation of the type strain from the jejunum).

The description is based on three strains isolated from the human jejunum. Cells are obligately anaerobic, non-motile, Gram-stain-negative bacilli (0.7×0.8–2 µm). After 3–5 days of incubation on blood agar plates, colonies are 1–2 mm in diameter, circular, convex, weakly to moderately pigmented and weakly to strongly haemolytic. The optimum conditions for growth are 37 °C and pH 6–7. Acetic acid, succinic acid and small amounts of isovaleric acid are produced from glucose. NH_3_ is produced. Cells are saccharolytic and proteolytic and are able to ferment glucose, lactose, maltose, mannose, raffinose and sucrose, but not arabinose, cellobiose, mannitol, melezitose, rhamnose, salicin, sorbitol, trehalose or xylose. Positive for activity of β-galactosidase, β-galactosidase-6-phosphate, α-glucosidase, *N*-acetyl-β-glucosaminidase, α-fucosidase, sialidase, acid phosphatase, alkaline phosphatase, naphthol-AS-BI-phosphate, arginine arylamidase, alanine arylamidase, leucine arylamidase and leucyl glycine arylamidase (Table S2). Gelatin is hydrolysed but aesculin is not. Cells agglutinate human AB and O erythrocytes and bind to several human intestinal cell lines. The predominant CFA is anteiso-C_15 : 0_, accounting for 42.5 % of the total CFA profile. 

The type strain is CD3 : 28^T^ ( = CCUG 60371^T^ = DSM 26989^T^), which was isolated from a biopsy of the small intestine of a child with CD. Strains CD3 : 27 ( = CCUG 60308) and CD3 : 33 ( = CCUG 60311) are additional strains of this species. The DNA G+C content of the type strain is 41.7 mol%.
